# Dauricine Attenuates Spatial Memory Impairment and Alzheimer-Like Pathologies by Enhancing Mitochondrial Function in a Mouse Model of Alzheimer's Disease

**DOI:** 10.3389/fcell.2020.624339

**Published:** 2021-02-05

**Authors:** Chongyang Chen, Pan Liu, Jing Wang, Haitao Yu, Zaijun Zhang, Jianjun Liu, Xiao Chen, Feiqi Zhu, Xifei Yang

**Affiliations:** ^1^Shenzhen Key Laboratory of Modern Toxicology, Shenzhen Medical Key Discipline of Health Toxicology (2020-2024), Shenzhen Center for Disease Control and Prevention, Shenzhen, China; ^2^Key Laboratory of Innovative Chemical Drug Research in Cardio-Cerebrovascular Diseases, Institute of New Drug Research and Guangzhou, Jinan University College of Pharmacy, Guangzhou, China; ^3^Cognitive Impairment Ward of Neurology Department, The Third Affiliated Hospital of Shenzhen University Medical College, Shenzhen, China

**Keywords:** Alzheimer's disease, dauricine, proteomics, mitochondrial function, synaptic function

## Abstract

Alzheimer's disease (AD) is characterized by extracellular amyloid plaques composed of β-amyloid (Aβ) and intracellular neurofibrillary tangles containing hyperphosphorylated tau protein. No effective therapy is available for this disease. In this study, we investigated the potential therapeutic effects of dauricine (DAU), a benzyl tetrahydroisoquinoline alkaloid, on AD, and found that DAU administration significantly improved cognitive impairments in 3xTg-AD mice by decreasing Aβ plaques and hyperphosphorylated tau and increasing the hippocampal ATP level. Proteomic and western blot analyses revealed that DAU treatment mainly modified the expression of proteins involved in mitochondrial energy metabolism, such as Aco2, Ndufs1, Cox5a, and SDHB, and that of synapse-related proteins such as Syn1 and Syn2. Pathway analysis revealed that DAU modulated the tricarboxylic acid cycle, synaptic vesicle cycle, glycolysis, and gluconeogenesis in 3xTg-AD mice. Our study suggests that DAU may be a potential drug for the treatment of AD.

## Introduction

Alzheimer's disease (AD) is an age-dependent neurodegenerative disease. Patients with AD show language obstacle, loss of motor capacity, and inability for self-care as the disease progresses (Burns and Iliffe, 2009). By 2050, the number of people aged 65 and older with AD may rise to 13.8 million (Hebert et al., 2013). Both, the initiation of AD and deterioration in AD, involve protein degradation, inflammation, mitochondrial dysfunction, and synaptic loss (Querfurth and Laferla, 2010; Bonet-Costa et al., 2016). However, single-target drugs designed for these mechanisms have failed in clinical trials (Frautschy and Cole, 2010; Golde et al., 2011). Thus, developing multiple-target drugs may be an alternative strategy, which is more promising for delaying the onset of AD.

Recently, natural compounds with multiple biological activities have aroused widespread interest in the field of AD (Andrade et al., 2019). Dauricine (DAU) is an isoquinoline alkaloid and is extracted from the rootstock of a traditional Chinese medicine, *Menispermum dauricum* DC. In ischemic models, it has been demonstrated that DAU protects neurons and inhibits cell apoptosis by regulating mitochondrial function (Li and Gong, 2007; Wang et al., 2020). In addition, DAU can ameliorate tau hyperphosphorylation (Wang et al., 2005) and promote β-amyloid (Aβ)_1−42_ clearance by releasing bradykinin (Pu et al., 2018). Our group has previously confirmed the neuroprotective effect of DAU on AD model cells (Liu et al., 2018). However, its beneficial effects and the underling mechanisms *in vivo* are unknown and require further investigation in AD animal models.

The present study aimed to investigate whether, and how, DAU could moderate AD pathologies and AD-related learning and memory deficits in an AD mouse model. We found that DAU administration significantly attenuated cognitive impairments in 3xTg-AD mice by decreasing Aβ plaques and hyperphosphorylated tau and increasing the hippocampal ATP level. Mechanically, DAU treatment mainly modified the expression of synapse-related proteins and proteins involved in the mitochondrial energy metabolism. Moreover, pathway analysis showed that DAU modulated glycolysis and gluconeogenesis in 3xTg-AD mice. Our study suggests that DAU may be a potential drug for the treatment of AD.

## Materials and Methods

### Reagents

We purchased DAU (stated purity ≥ 98%) from Shanghai Aladdin Biochemical Technology (CAS: 524-17-4, D115683, Shanghai, China) and have listed the antibodies in Supplementary Table 1.

### Treatment of Experimental Animals

Triple transgenic AD mice (3xTg-AD; strain: APPSwe, PS1M146V, and TauP301L) and wild-type (WT) mice (strain: B6129SF2/J) were purchased from the Jackson Laboratory (Maine, USA). We intraperitoneally injected 8 month-old female transgenic and wild mice with DAU (1 or 10 mg/kg) and saline of equivalent volume, respectively, for 2 months. Each group comprised 13 mice, and DAU dose was based on a previous study (Jin et al., 2010). The mouse age was based on the pathological stages of 3xTg-AD mice (Oddo et al., 2003). After 2 months of DAU treatment, cognitive abilities of all the animals were assessed. All animals were sacrificed after behavioral tests, for further studies. Animal experiments and manipulation were approved by the Shenzhen Center for Disease Control and Prevention. We made efforts to minimize animal suffering and reduce the number of mice used.

### Behavioral Test

#### Step-Down Passive Avoidance Test

After DAU administration, short-term learning and memory of all mice were evaluated with the step-down passive avoidance test, as previously reported (Kameyama et al., 1986; Zhou et al., 2019). For the training test, each mouse was gently placed on a platform and an electrical shock (36 V) was delivered through grid floor for 5 min. After 24 h, the training and retention tests were performed. Each mouse was placed on the platform and the electrical shock was delivered and all the data were recorded for analysis.

#### Morris Water Maze Test

After step-down passive avoidance test, hippocampal-dependent spatial learning and memory of all mice were evaluated using the Morris water maze (MWM) test. In brief, mice were trained for 5 consecutive days to find a platform in the water maze. During the training session, each mouse was placed into water, facing the wall, starting from one of the four quadrants, and a computer tracking program was started. The swim time was set to 60 s. The timer was paused once the mouse climbed on the platform. Thereafter, the mouse was removed from the pool. Alternatively, the mouse was placed on the platform for an additional 15 s. The probe trial was performed 6 days after the training session. The platform was removed, and the mouse was allowed to swim freely for 2 min before being removed from the pool. The tracks were recorded by a computer program.

### Proteomic Analysis

#### Protein Preparation and Labeling

After behavioral tests, mice were sacrificed for sample collection. The protocol of proteomic analysis has been reported previously (Huang et al., 2018). Half of the mice hippocampus were lysed in 500 μL DIGE specific lysis buffer for 30 min on ice and centrifuged at 12,000 g for 20 min at 4°C. Thereafter, the supernatant was collected. Each sample supernatant was mixed with lysis buffer to remove salt using a centrifugal filter. After protein quantification, each protein sample was diluted to a final concentration of 5 μg/μL. Thereafter, the sample (5 μL) was labeled with Cy3 or Cy5 in the dark for 30 min. Samples pooled from each group were stained by Cy2, the internal standard. Finally, protein labeling was terminated by adding 10 mM lysine. The labeled protein was mixed into a group and rehydration buffer and 0.002% bromophenol blue were added. Thereafter, they were transferred onto immobilized pH gradient strips.

#### 2-Dimensional Electrophoresis

Briefly, after isoelectric-focusing (IEF), the strips were equilibrated and loaded on the top of 12.5% SDS-PAGE gels. Thereafter, protein separation in the second dimension was performed using an Etan DALTsix electrophoresis system at 15°C. The gels were run at 1 W/gel for 1 h followed by 11 W/gel for 6 h. Finally, gels were scanned using a Typhoon TRIO Variable Mode Imager, with resolution set at 100 μm for image acquisition.

#### Image Analysis

The DeCyder software package was used for DIGE gel analysis. Briefly, the gel image was imported into the software and processed with the Differential In-gel Analysis. Each protein spot in the Cy3 or Cy5 channel was normalized to the corresponding spot in the Cy2 channel. The differentially expressed protein spots (p < 0.05) were isolated for further study.

#### In-gel Digestion by Trypsin and Mass Spectrometry

A total of 1-mg protein sample was used for in-gel digestion. In brief, after staining the gels, the differentially expressed spots were manually isolated from the stained gel. A piece from each gel was transferred into a 1.5 mL tube and digested with trypsin at 37°C overnight. The digested peptides were used for mass spectrometry (MS) analysis using an AB SCIEX MALDI-TOF/TOF 5800 mass spectrometer. MASCOT (Matrix Science, UK) was used for database searching against the SwissProt mouse protein database.

#### Bioinformatics Analysis

The Database for Annotation, Visualization, and Integrated Discovery (DAVID) Bioinformatics Resources 6.8 was used for functional enrichment analysis. Pathway analysis was performed using Wikipathways (https://www.wikipathways.org/) and Kyoto Encyclopedia of Genes and Genomes (KEGG) pathway database (https://www.kegg.jp/kegg/). The protein–protein interaction (PPI) network analysis was visualized using Cytoscape 3.7.1 software.

### Western Blot Analysis

The sample was lysed with RIPA lysis buffer. The protein concentration was measured by the BCA method. The sample supernatant was mixed with loading buffer and heated for 10 min at 100°C. Thereafter, samples were separated on an 8–12% SDS–PAGE gel, transferred onto a PVDF membrane, and blocked with 5% skim milk. After blocking, the membranes were incubated with primary antibodies (Supplementary Table 1) followed by a secondary antibody. Finally, the membranes were exposed with enhanced chemiluminescence kit and protein quantification was performed using ImageJ software.

### Immunohistochemistry Analysis

The mouse brain sections embedded in paraffin were deparaffinized and rehydrated by xylene treatment followed by gradual ethanol treatment (100–70%). Thereafter, the sections were blocked and incubated with a primary antibody, AT8 or 6E10, at 4°C overnight. After incubation with primary antibody, the sections were stained using a DAB kit. The images were observed under a microscope (Olympus BX60, Tokyo, Japan). We used Image-pro plus 6.2 for image quantify analysis.

### Detection of the ATP Level

The ATP assay kit (Beyotime, Haimen, China) was used to detect the ATP level in hippocampal tissues. Briefly, samples were extracted using ATP lysis buffer and quantified by the BCA method. The reaction mixture, containing either the sample or standard (100 μL) with ATP detection fluid (100 μL), was incubated at room temperature for 3–5 min. A microplate reader with luminometer function was used to measure the ATP level. The ATP level was calculated as nmol/mg protein.

### Statistical Analysis

Data are presented as mean ± standard error of the mean (SEM). Statistical analyses were performed using ANOVA (equal variance) or Welch's ANOVA (unequal variance). SPSS 21.0 software was used for data analysis. A *p* < 0.05 indicated significant difference among the groups.

## Results

### DAU Treatment Ameliorated Cognitive Impairment in 3xTg-AD Mice

**Figure 1:**
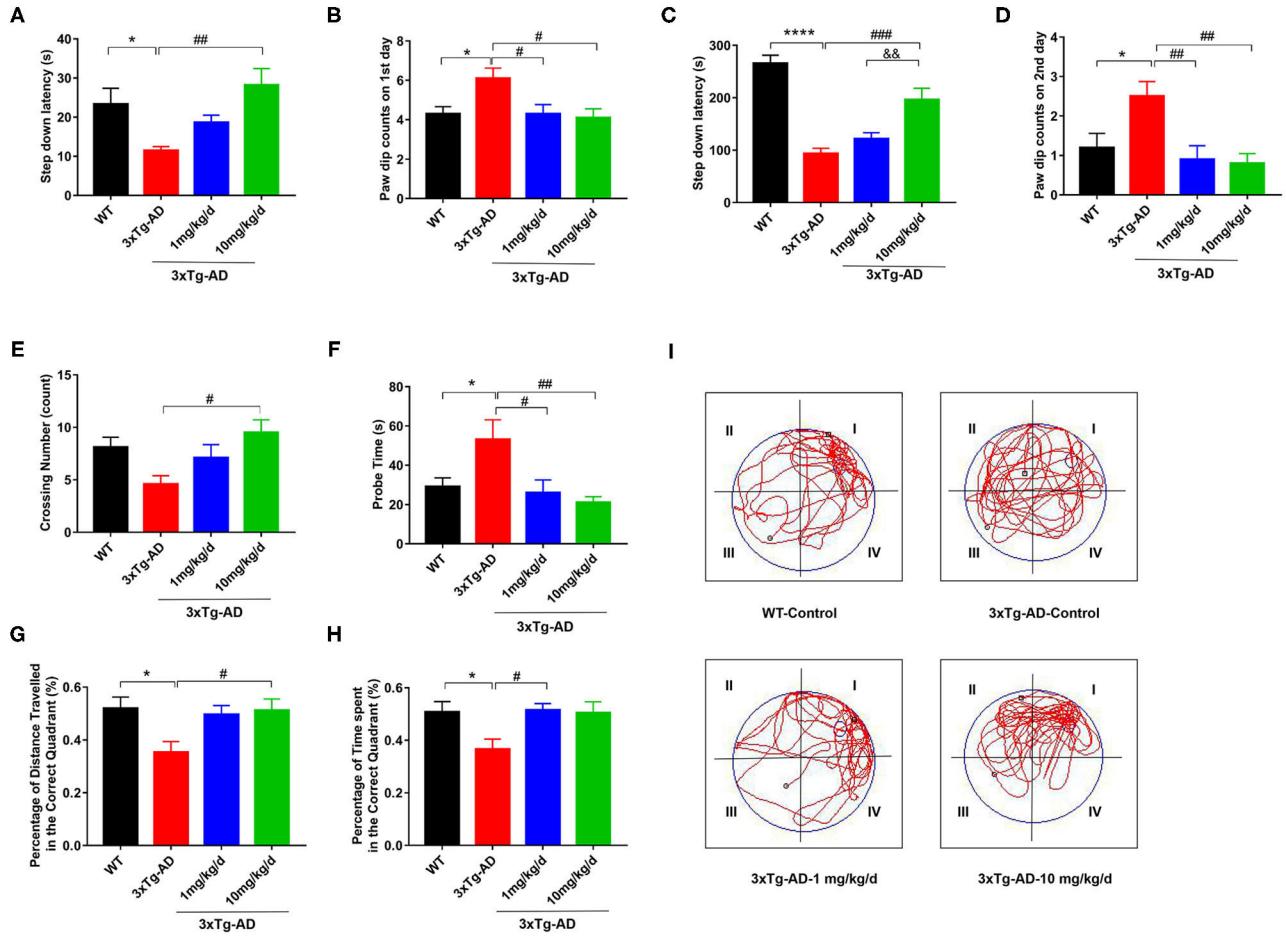
DAU improves the spatial learning and memory abilities of 3xTg-AD mice. **(A,B)** The latency in the retention test and the number of errors on the first day. **(C,D)** The latency in the retention test and the number of errors on the second day. **(E–I)** Morris water maze (MWM) test. **(E)** Crossing number, **(F)** probe time, **(G)** the percentage of distance traveled in the correct quadrant, **(H)** the percentage of time spent in the correct quadrant, and **(I)** the representative swimming path. Data are presented as mean ± SEM. *p < 0.05, ****p < 0.0001 vs. WT saline group. ^#^*p* < 0.05, ^##^*p* < 0.01, ^###^*p* < 0.001 vs. 3xTg-AD saline group. ^&&^*p* < 0.01 vs. 3xTg-AD-1 mg/kg/d group. *N* = 10–14 for each group.

First, we treated 8 month-old 3xTg-AD mice with DAU for 2 months and employed step-down passive avoidance and MWM tests to evaluate the effect of DAU on cognitive function. We found that compared with WT mice, 3xTg-AD mice had a shorter step-down latency and more paw dip counts during the 2 test days, indicating memory deficits in 3xTg-AD mice (Figures 1A–D). Interestingly, treatment with 10 mg/kg/d DAU significantly improved cognitive capacity of 3xTg-AD mice, as evidenced by increased step-down latency and less paw dip counts (Figures 1A–D). However, treatment with 1 mg/kg/d DAU could only rescue the error numbers and paw dip counts were unchanged (Figures 1A–D), indicating partial efficiency at low dosage. Thereafter, we performed MWM test to further evaluate the effect of DAU on spatial memory improvement. No difference was found among the groups during the 5 day training (Supplementary Figure 1). However, in the probe trial, DAU treatment at both low and high dosages resulted in robust rescue of memory dysfunction (Figures 1E–I). Overall, these data suggested that DAU treatment can improve cognitive capacity of 3xTg-AD mice.

### DAU Treatment Reduced Aβ Accumulation and Tau Hyperphosphorylation

**Figure 2:**
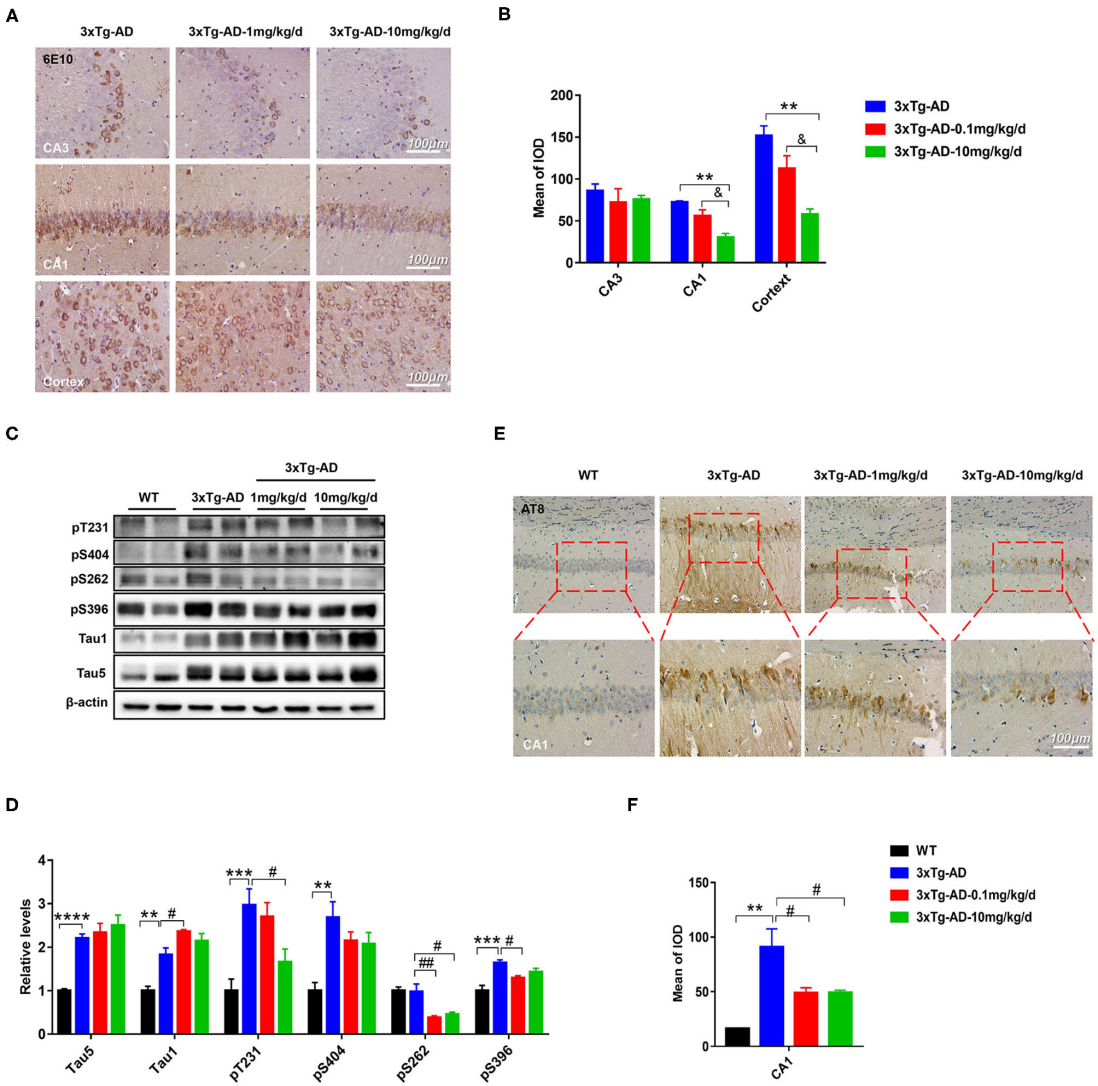
DAU reduced the levels of Aβ and hyperphosphorylated tau in 3xTg-AD mice. **(A,B)** Immunohistochemical staining of the CA1, CA3, and cortex of 3xTg-AD mice with 6E10 and quantitative statistics of positive staining (*n* = 3 per group). **(C,D)** Western blot analysis for the expression level of pT231, p404, p262, p396, Tau1, and Tau5 (*n* = 4 per group). **(E,F)** Immunohistochemical staining of the CA1 region of 3xTg-AD with AT8 and quantitative statistics of positive staining (*n* = 3 per group). Data are presented as mean ± SEM. **p < 0.01, ***p < 0.001, ****p < 0.0001 vs. WT vehicle group. ^#^*p* < 0.05, ^##^*p* < 0.01 vs. 3xTg-AD saline group. ^&^*p* < 0.05 vs. 3xTg-AD-1mg/kg/d group.

Extracellular amyloid plaques of Aβ and intracellular neurofibrillary tangles of hyperphosphorylated tau protein are the two characteristic pathologies in AD brain. Both of them contribute to the cognitive disorder in patients with AD. To explore the effect of DAU on these pathologies, we used 6E10 antibody, which recognizes 1–16 amino acids of Aβ, and AT8 antibody, which recognizes PHF-tau at Ser202/Thr205. Immunohistochemical staining revealed that Aβ accumulation in the hippocampal CA1 and cortical regions was significantly reduced after DAU treatment (10 mg/kg/d) (Figures 2A,B). Meanwhile, the positive staining of AT8 significantly reduced in the hippocampal CA1 region (Figures 2E,F). Further, Western blot analysis was performed to detect tau phosphorylation at serine 404, 262, and 396, and threonine 231 or unphosphorylated tau (Tau1) (Figures 2C,D). In 3xTg-AD mice, compared with vehicle-injected mice, DAU treatment both at 1 and 10 mg/kg/d significantly decreased the level of tau hyperphosphorylation. Interestingly, reversal of tau hyperphosphorylation at pT231 was more efficient with 10 mg/kg/d DAU than 1 mg/kg/d DAU. These data indicated that DAU treatment is beneficial in AD pathologies.

### Proteomic Analysis Revealed That DAU Treatment Improved Mitochondrial Function

**Figure 3:**
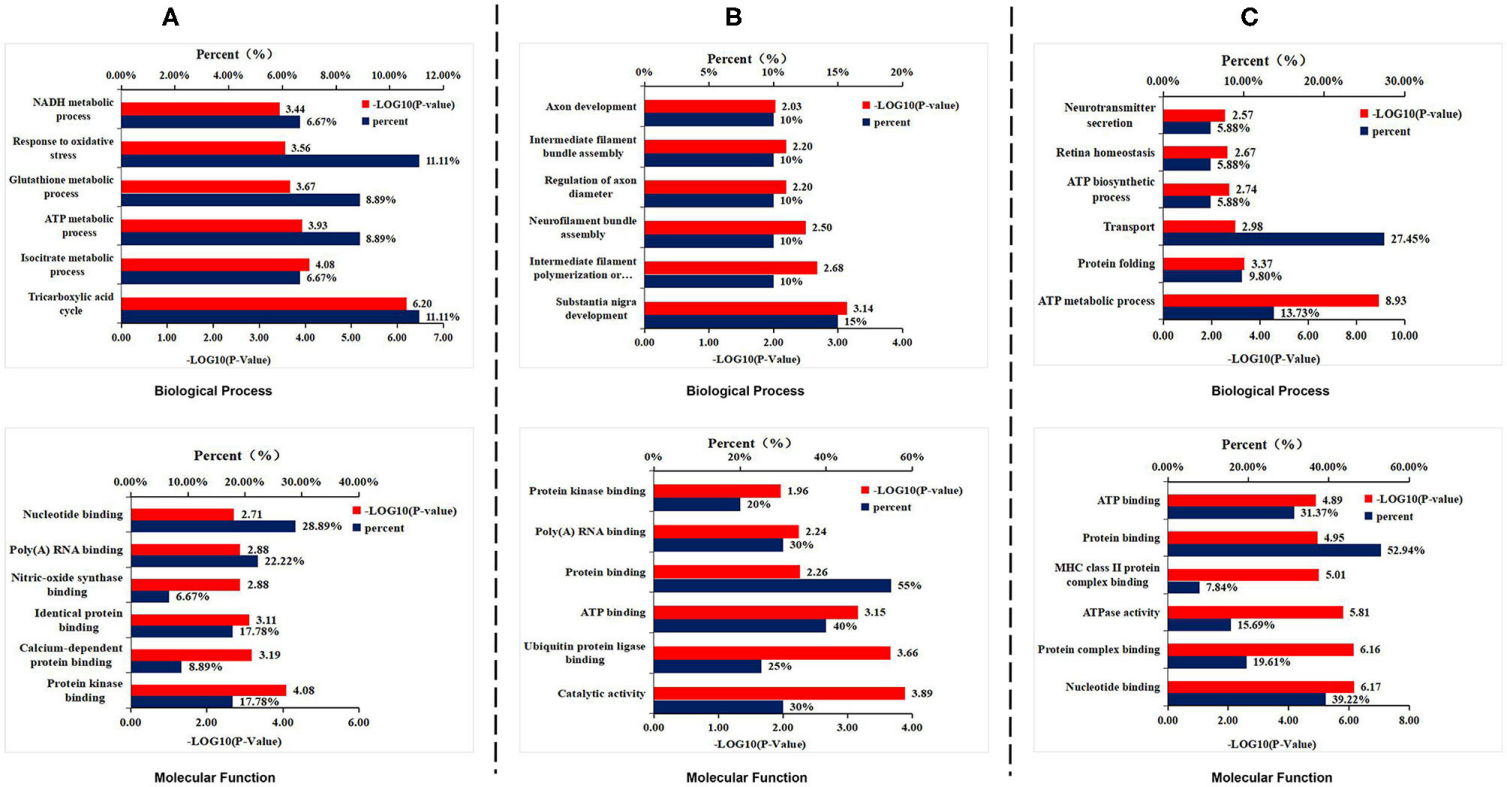
Gene Ontology enrichment analysis for the differentially expressed proteins. **(A)** The biological process and molecular function analyses of the differentially expressed proteins in 3xTg-AD mice. **(B)** 1 mg/kg/d DAU-treated 3xTg-AD mice and **(C)** 10 mg/kg/d DAU-treated 3xTg-AD mice.

To explore how DAU treatment ameliorated AD pathologies and improved spatial memory, 2D-DIGE separation and MS identification were performed to detect protein changes in the hippocampus of 3xTg-AD mice. We found that 45 proteins were differentially expressed in WT mice compared with 3xTg-AD mice, 51 proteins in 3xTg-AD treated with 1 mg/kg/d DAU compared with 3xTg-AD mice, and 20 proteins in 3xTg-AD mice treated with 10 mg/kg/d DAU compared with 3xTg-AD mice (Supplementary Table 2). Heat maps present the change in expression of differentially expressed proteins in different mouse groups (Supplementary Figure 2). DAVID was used to determine the biological process and molecular functions of differentially expressed proteins (Figures 3A–C). Biological process analysis revealed that differentially expressed proteins were mainly enriched in the tricarboxylic acid (TCA) cycle, ATP metabolic process, glutathione metabolic process, and oxidative stress response process in 3xTg-AD mice, compared with WT mice. Molecular function analysis revealed that these proteins were mainly enriched in nucleotide binding, poly(A) RNA binding, and protein kinase binding (Figure 3A). Similarly, differentially expressed proteins were found to be enriched in substantia nigra development, intermediate filament polymerization or depolymerization, neurofilament bundle assembly, regulation of axon diameter, intermediate filament bundle assembly, and axon development by biological process analysis and in ATP binding, protein binding, and poly(A) RNA binding (Figure 3B) by molecular function analysis in 3xTg-AD mice treated with 1 mg/kg/d DAU compared with vehicle-treated 3xTg-AD mice. Further, we found that DAU treatment (10 mg/kg/d) resulted in the enrichment of these proteins in the ATP metabolic process, protein folding and transport, ATP biosynthesis, retina homeostasis, and neurotransmitter secretion by biological process analysis, and in nucleotide binding, protein binding, and ATP binding by molecular function analysis (Figure 3C). DAVID annotation revealed that the proteins differentially expressed in 3xTg-AD mice, compared with WT mice, were mainly involved in mitochondrial energy metabolism, suggesting a role of mitochondrial dysfunction in AD progression. However, DAU treatment not only rescued abnormal mitochondrial energy metabolism but also improved synaptic function. These results indicated that modifications in mitochondrial function and synaptic function may contribute to the beneficial effects of DAU treatment on AD.

**Figure 4:**
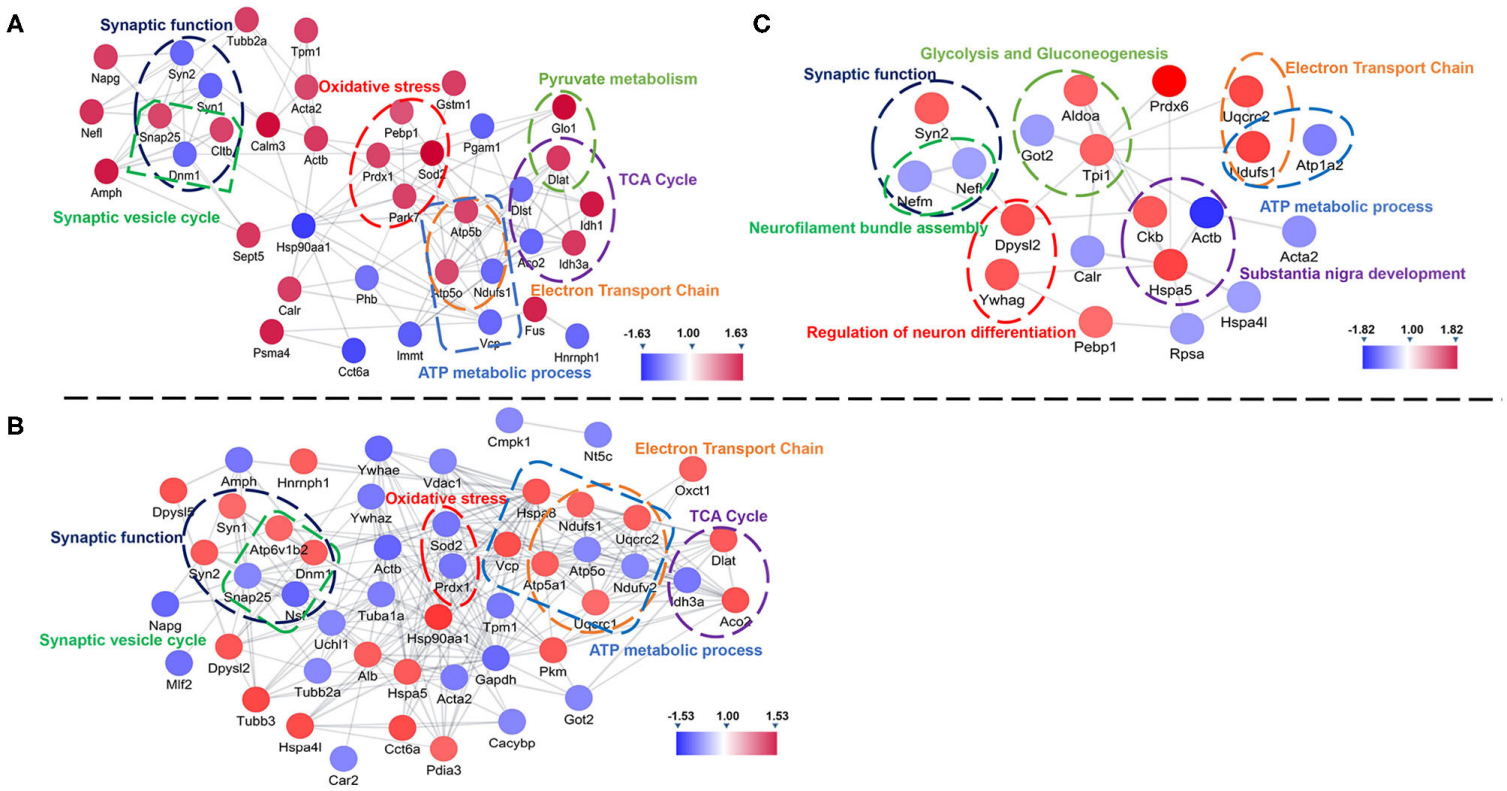
Protein–protein interaction analysis of the differentially expressed proteins using the STRING database. **(A)** The protein–protein interaction (PPI) maps of the differentially expressed proteins in untreated 3xTg-AD mice vs. WT mice. **(B)** The PPI maps of the differentially expressed proteins in 1 mg/kg/d DAU-treated 3xTg-AD mice vs. untreated 3xTg-AD mice. **(C)** The PPI maps of the differentially expressed proteins in 10 mg/kg/d DAU-treated 3xTg-AD mice vs. untreated 3xTg-AD mice. Visualization of PPI maps in Cytoscape 3.7.1 software: green represents downregulation and red represents upregulation.

**Figure 5:**
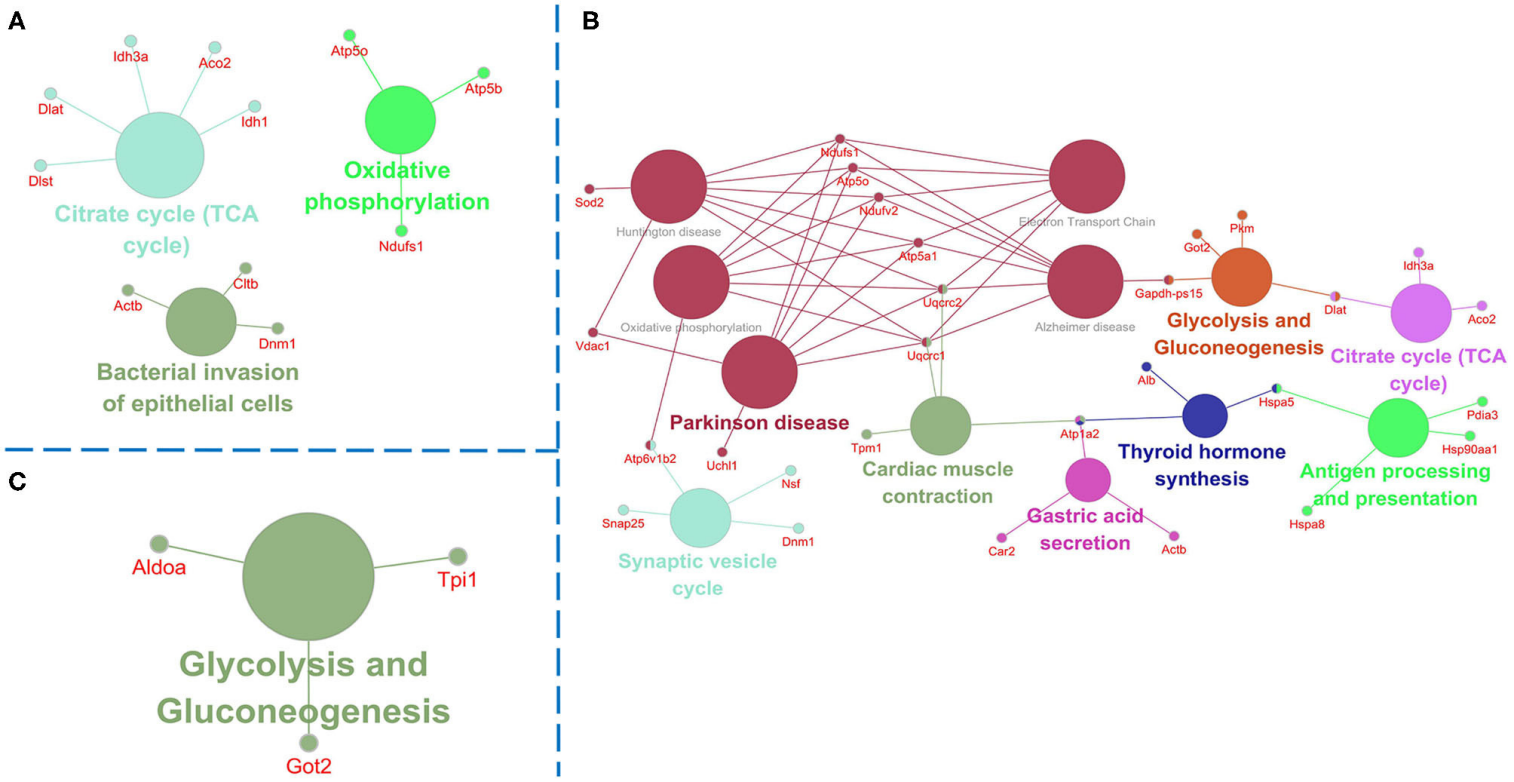
Pathway analysis of the differentially expressed proteins. Pathway analysis using Clue GO software and the *p*-value was set at <0.05. **(A)** Comparison of pathways of the dysregulated proteins between untreated 3xTg-AD mice and WT mice. **(B)** Comparison of pathways of the dysregulated proteins between 1 mg/kg/d DAU-treated 3xTg-AD mice and untreated 3xTg-AD mice. **(C)** Comparison of pathways of the dysregulated proteins between 10 mg/kg/d DAU-treated 3xTg-AD mice and untreated 3xTg-AD mice.

Further, we used String and Wiki pathway databases to analyze PPIs and delineate enriched signaling pathways (Figures 4, 5). The interaction of differential proteins was enriched in synaptic function, pyruvate metabolism, oxidative stress response, and the synaptic vesicle cycle, ATP metabolic process, electron transport chain, and TCA cycle in 3xTg-AD mice compared with WT mice (Figure 4A). DAU treatment (1 or 10 mg/kg/d) distinctly changed proteins in the pathway of mitochondrial energy metabolism (electron transport chain, glycolysis, and gluconeogenesis) and synaptic function (synaptic vesicle cycle; Figures 4B,C). The PPI analysis revealed that the differentially expressed proteins were mainly enriched in mitochondrial energy metabolism. Additionally, the expression level of the same proteins in other mouse groups, showed in PPI maps, were reversed after DAU treatment. These include synaptic function proteins (Synapsin-1 [Syn1], Synapsin-2 [Syn2]) and mitochondrial energy metabolism proteins (NADH-ubiquinone oxidoreductase 75 kDa subunit [Ndufs1], ATP synthase subunit O [ATP5o], isocitrate dehydrogenase [NAD] subunit alpha [Idh3a], aconitate hydratase [Aco2]).

Pathway analysis showed that differentially expressed proteins were mainly involved in pathways related to mitochondrial energy metabolism (Figure 5). Briefly, compared with WT mice, 3xTg-AD mice showed pathways in TCA cycle, oxidative phosphorylation and bacterial invasion of epithelial cells. Treatment with 1 mg/kg/d DAU had an effect on pathways mainly associated with Parkinson disease, glycolysis, gluconeogenesis, the TCA cycle, and the synaptic vesicle cycle. However, treatment with 10 mg/kg/d DAU had an effect only on glycolysis and gluconeogenesis. Thus, we focused on mitochondrial energy metabolism and used Cytoscape software to determine the detailed expression change of proteins in the TCA cycle and electron transport chain in mitochondrial energy metabolism. The TCA cycle and electron transport chain in 3xTg-AD mice vs. WT mice are presented in Supplementary Figures 3A,B, and those in 3xTg-AD-1 mg/kg/d mice vs. 3xTg-AD mice are presented in Supplementary Figures 3C,D. Pathway analysis revealed that Aco2, Ndufs1, mitochondrial cytochrome b-c1 complex subunit 1 (Uqcrc1), mitochondrial cytochrome b-c1 complex subunit 2 (Uqcrc2), and mitochondrial ATP synthase subunit alpha (ATP5a1) proteins were upregulated after 1 mg/kg/d DAU treatment. Similarly, at 10 mg/kg/d DAU dose, the mitochondrial proteins (Ndufs1 and Uqcrc1) and glycolysis and gluconeogenesis proteins (fructose-bisphosphate aldolase A [Aldoa] and triosephosphate isomerase [Tpi1]) were upregulated after treatment (Supplementary Table 2). Moreover, the expression level of Aco2, the key enzyme for the conversion of citrate to isocitrate (ICT; an important biological process in the TCA cycle), was reversed in DAU-treated 3xTg-AD mice compared with vehicle-treated 3xTg-AD mice. These results indicated that DAU treatment may reverse the defects of mitochondrial energy metabolism in 3xTg-AD mice and benefit mitochondrial function. In addition, synaptic proteins (Syn1 and Syn2) were upregulated, whereas oxidative stress protein (mitochondrial superoxide dismutase [Mn-Sod2] and Peroxiredoxin-1[Prdx1]) were downregulated after DAU treatment. The reversed expression of Syn1 and Syn2 in DAU-treated 3xTg-AD mice revealed synaptic protection.

### Western Blot Analysis and Functional Detection Proved That DAU Treatment Ameliorated Mitochondrial Function in 3xTg-AD Mice

**Figure 6:**
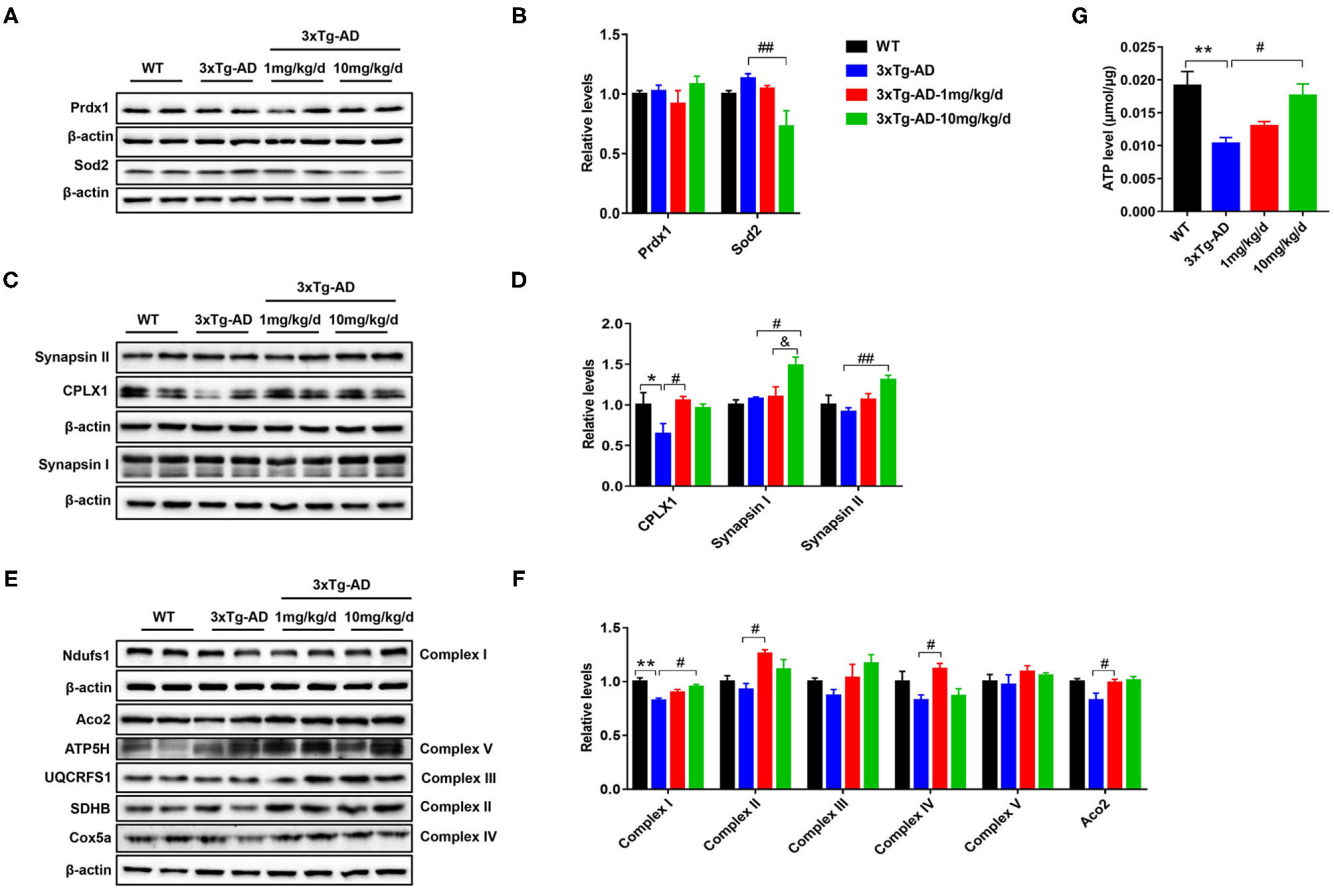
Verification of protein dysregulation and measurement of electron transport chain protein and hippocampal ATP levels. **(A,B)** The changes in the expression levels of oxidative stress proteins—Prdx1 and Sod2. **(C,D)** The expression levels of synaptic function proteins—CPLX1, Synapsin I, and Synapsin II. **(E,F)** The change in the expression levels of Aco2 and election transport chain proteins—Ndufs1, SDHB, UQCRFS1, Cox5a, and ATP5H. **(G)** Hippocampal ATP levels in each group. The data are presented as mean ± SEM. *p < 0.05, **p < 0.01 vs. WT saline group. ^#^*p* < 0.05, ^*##*^*p* < 0.01 vs. 3xTg-AD saline group. ^&^*p* < 0.05 vs. 3xTg-AD-1 mg/kg/d group. *N* = 4 for each group.

To further confirm the differentially expressed proteins screened by proteomics, we performed western blot analysis to measure the altered concentrations of Prdx1, Sod2, Syn1, Syn2, Aco2, and Ndufs1 proteins. Compared with untreated 3xTg-AD mice, the expression level of Sod2 was significantly decreased, whereas the expression levels of Syn1, Syn2, and Ndufs1were significantly increased in 10 mg/kg/d DAU-treated 3xTg-AD mice. Moreover, the expression level of Aco2 was significantly increased in 1 mg/kg/d DAU-treated 3xTg-AD mice (Figures 6A–F). Further, we measured the expression of electron transport chain proteins (mitochondrial succinate dehydrogenase [ubiquinone] iron-sulfur subunit [SDHB-complex II], mitochondrial cytochrome b-c1 complex subunit Rieske (UQCRFS1-complex III), mitochondrial cytochrome c oxidase subunit 5A [Cox5a-complex IV], mitochondrial ATP synthase subunit d [ATP5H-complex V]), and the ATP level in 3xTg-AD mice to evaluate the beneficial effects of DAU on mitochondria. The expression levels of Aco2, SDHB-complex II, and Cox5a-complex IV proteins were significantly increased in 1 mg/kg/d DAU-treated 3xTg-AD mice, whereas in 10 mg/kg/d DAU-treated 3xTg-AD mice, the expression level of Ndufs1-complex I was significantly increased, UQCRFS1-complex III and SDHB-complex II showed an upregulated trend (Figures 6E,F). However, the ATP level was only significantly increased in 10 mg/kg/d DAU-treated 3xTg-AD mice (Figure 6G). These data indicated that DAU indeed improved the mitochondrial function in 3xTg-AD mice, especially at 10 mg/kg/d dosage. The upregulation of Syn1 and Syn2 proteins in 3xTg-AD mice after DAU treatment indicated that DAU may simultaneously modulate synaptic function in AD progression.

## Discussion

DAU, a bisbenzylisoquinoline alkaloid, is isolated from dauricum DC, and can treat various diseases such as cardiac ischemia, angina, and inflammation, as well as inhibit angiogenesis in tumors and promote apoptosis in tumor cells (Xia et al., 2002; Tang et al., 2009; Yang et al., 2010). Our previous study showed that DAU treatment attenuated hyperphosphorylation of tau and production of Aβ in N2a/APP cells (Liu et al., 2018). In this study, we further confirmed that DAU can improve cognitive impairment and neurodegeneration in 3xTg-AD mice. Treatment with 10 mg/kg/d DAU can significantly improve the learning and memory ability of AD mice in step-down passive avoidance and MWM tests and decrease Aβ (6E10) and phosphorylated tau (AT8) positive staining. Moreover, Western blot analysis showed that DAU treatment could reduce phosphorylated tau at the dose of 1 mg/kg/d. Pathological changes of Aβ and tau proteins are consistent with the findings of our previous *in vitro* study (Liu et al., 2018). In the *in vitro* study, DAU treatment inhibited APP processing to reduce Aβ production and attenuated tau pathology through PP2A and p35/25, and the main mechanism may have involved antioxidant stress. The homeostatic health of mitochondria contributed to the attenuation of Aβ and tau pathologies (Fang et al., 2019). In this study, it was found that DAU treatment improved mitochondrial function, and that the antioxidant effect may benefit the attenuation of tau phosphorylation and Aβ production.

Proteomics analysis revealed that the biological functions of differentially expressed proteins in three different mouse groups mainly involved mitochondrial energy metabolism. Consistent with the biological function, the differentially express proteins were enriched in the following signaling pathways: the TCA cycle, oxidative phosphorylation, glycolysis, and gluconeogenesis. In terms of mitochondrial energy metabolism, DAU treatment increased the expression of Ndufs1-complex I, SDHB-complex II, and Cox5a-complex IV, and modified the expression of the Ndufs1-complex I and ATP5o- complex V of electron transport chain in 3xTg-AD mice. In DAU-treated 3xTg-AD mice, the expression levels of Aco2 and Idh3a in the TCA cycle were modified. In agreement with these findings, the functional analysis showed an increase in the hippocampal ATP level in 3xTg-AD mice, suggesting that DAU improved mitochondrial energy metabolism. Moreover, DAU treatment increased the expression of Aco2—an enzyme that catalyzes the interconversion of citrate to ICT via cis-aconitate in the second step of the TCA cycle, and is essential for normal cell metabolism (Robbins and Stout, 1989; Gardner et al., 1995). Compared with the WT mice, the expression of Aco2 was decreased and the down-stream protein Idh3a, a catalytic subunit of the enzyme which catalyzes the decarboxylation of ICT into alpha-ketoglutarate, was increased in 3xTg-AD mice. Therefore, decreased expression of Aco2 and abnormally increased expression of Idh3a suppressed the TCA cycle and thus, the function of electron transport chain and energy metabolism. In contrast, DAU treatment rescued the mitochondrial energy metabolism deficit.

In the TCA cycle, the expression level of Aco2, an upstream independent regulatory enzyme, was different in DAU-treated and vehicle-treated 3xTg-AD mice. Idh3a is only a subunit of Idh3, a catalytic enzyme. Because DAU administration modified the expression of Aco2 in 3xTg-AD, and the TCA cycle being an upstream and important process of energy metabolism, Aco2 may be the key target of DAU treatment for regulating mitochondrial energy metabolism. A previous study revealed that Aco2 expression and activity were decreased in the peripheral blood of patients with AD and MCI (Mangialasche et al., 2015), which is consistent with our observation in this study. Additionally, Aco2 was sensitive to increased oxidative stress, which inactivated Aco2 activity (Tabrizi et al., 1999). DAU treatment decreased the expression of Sod2, resulting in increased ROS levels (De Leo et al., 1998). Sod2, an important enzyme that regulates mitochondrial ROS, and is closely related to mitochondrial function (Brand, 2010; Cox et al., 2018), was decreased in 3xTg-AD mice. DAU treatment modified the expression of Sod2 in 3xTg-AD mice, compared with WT mice. Thus, these data indicated that DAU not only improved mitochondrial function but also exerted antioxidant protective effects.

The energy supply is essential for synaptic activity (Harris et al., 2012). DAU treatment moderated the expression level of proteins involved in the synaptic vesicle cycle in 3xTg-AD mice. DAU administration reversed the expression level of Synaptosomal-associated protein 25 (Snap25)—a protein involved in the molecular regulation of neurotransmitter release (Blasi et al., 1993) and Dynamin-1 (Dnm1), in producing microtubule bundles, and in synaptic vesicle endocytosis (Raimondi et al., 2011). Moreover, Syn1 and Syn2 are involved in the regulation of neurotransmitter secretion (Rosahl et al., 1995), and Syn1 deficiency increases seizure propensity (Li et al., 1995) and alters short-term synaptic plasticity (Rosahl et al., 1993). Our result showed that DUA treatment increased the expression levels of Syn1, Syn2, and CPLX1, which positively regulated synaptic vesicles (Cao et al., 2013). Improved mitochondrial energy metabolism and upregulated synaptic proteins suggested that defects in synaptic function could be prevented by DAU treatment in 3xTg-AD mice.

**Figure 7:**
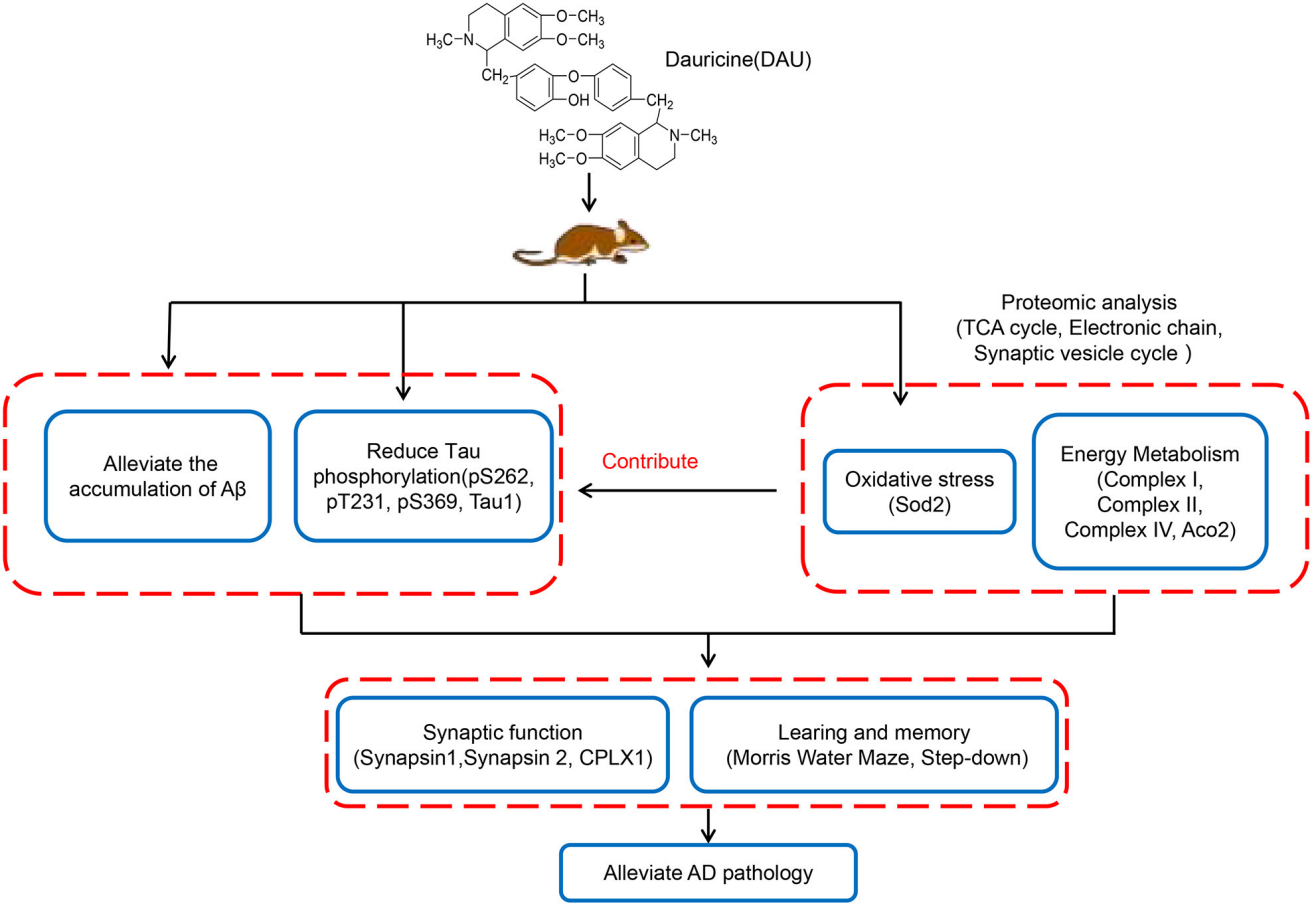
Hypothetical mechanisms of action of DAU.

In summary, this study revealed that DAU can significantly improve learning and memory impairment and AD-like pathologies. The mechanism of action of DAU may be the improvement of mitochondrial function and the modification of some key mitochondrial proteins, synaptic proteins, and antioxidant proteins in AD mice (Figure 7). Our data suggest DAU has the potential to be developed for the treatment of AD.

## Data Availability Statement

The original contributions presented in the study are included in the article/Supplementary Materials, further inquiries can be directed to the corresponding author/s.

## Ethics Statement

The animal study was reviewed and approved by Regulations for Animal Care and Use from the Committee of the Experimental Animal Center at Shenzhen Center for Disease Control and Prevention in Shenzhen, Guangdong Province, China.

## Author Contributions

CC and PL drafted the manuscript, performed the experiments, and analyzed the data. JW, HY, ZZ, JL, XC, FZ, and XY designed the study and analyzed the data. FZ and XY revised the manuscript. All authors contributed to the article and approved the submitted version.

## Conflict of Interest

The authors declare that the research was conducted in the absence of any commercial or financial relationships that could be construed as a potential conflict of interest.

## Supplementary Material

Supplementary Figure 1 The latency of mice in the water maze test after training for 5 consecutive days.

Supplementary Figure 2 Heat maps of the three mouse groups. **(A–C)** Heat map of the dysregulated proteins in different mouse groups. The rows represent proteins and columns represent groups. Red color indicates high abundance and blue color indicates low abundance.

Supplementary Figure 3 The TCA cycle and electron transport chain. **(A,B)** Comparison of the differentially expressed proteins associated with the TCA cycle and electron transport chain between untreated 3xTg-AD and WT mice and **(C,D)** between 1 mg/kg/d DAU-treated 3xTg-AD and untreated 3xTg-AD mice. Blue indicates downregulation and red indicates upregulation.

Supplementary Table 1 Sources and dilution ratios of the antibodies used for immunoblotting.

Supplementary Table 2 Differentially expressed proteins in DAU-treated vs. untreated 3xTg-AD mice.
