# Supplementary figures and images for: Dauricine Attenuates Spatial Memory Impairment and Alzheimer-Like Pathologies by Enhancing Mitochondrial Function in a Mouse Model of Alzheimer's Disease
Source: Front Cell Dev Biol. 2021 Feb 5;8:624339. doi: 10.3389/fcell.2020.624339 (PMC7902075; doi:10.3389/fcell.2020.624339)

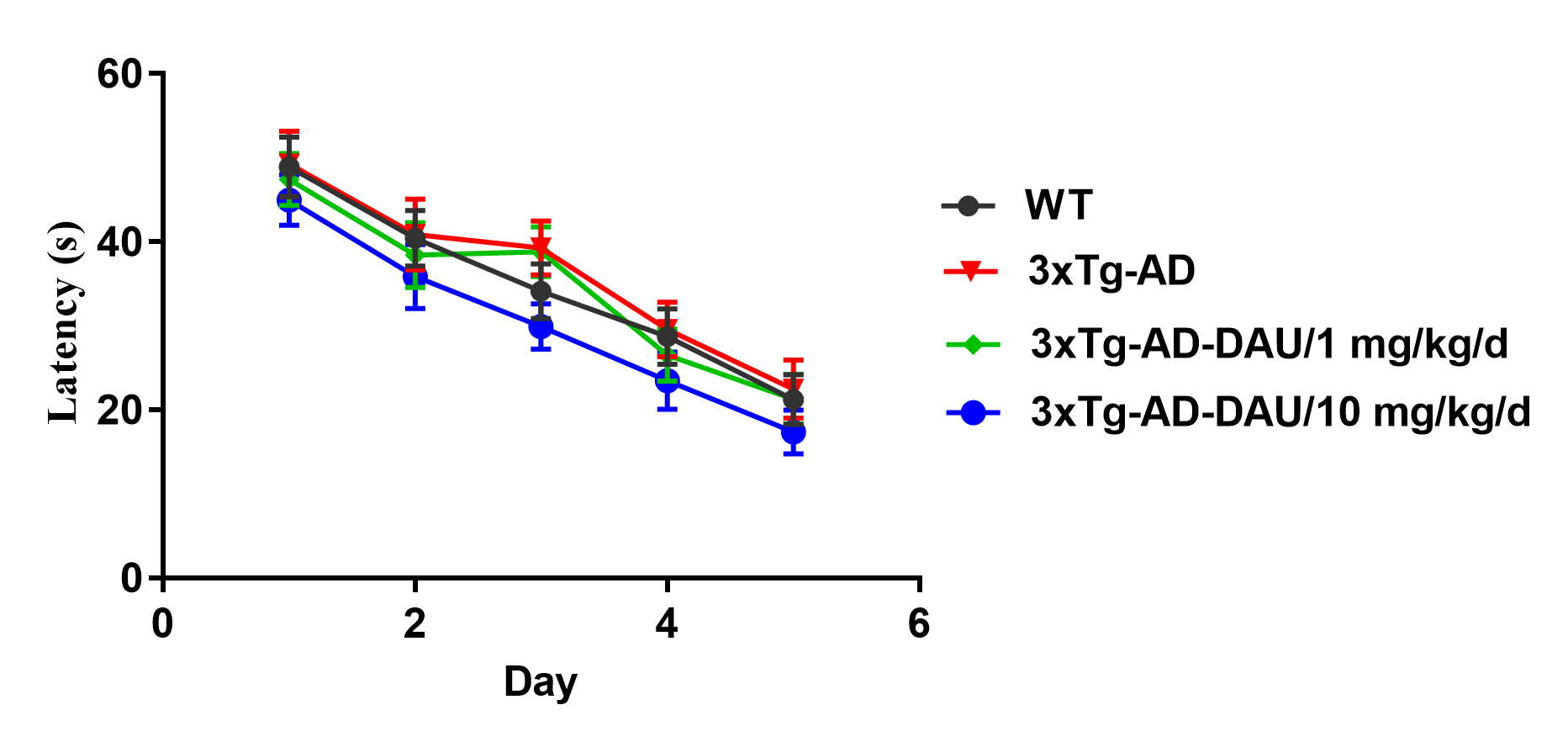

Supplement: Supplementary Figure 1 — The latency of mice in the water maze test after training for 5 consecutive days. [file Image_1.TIF]

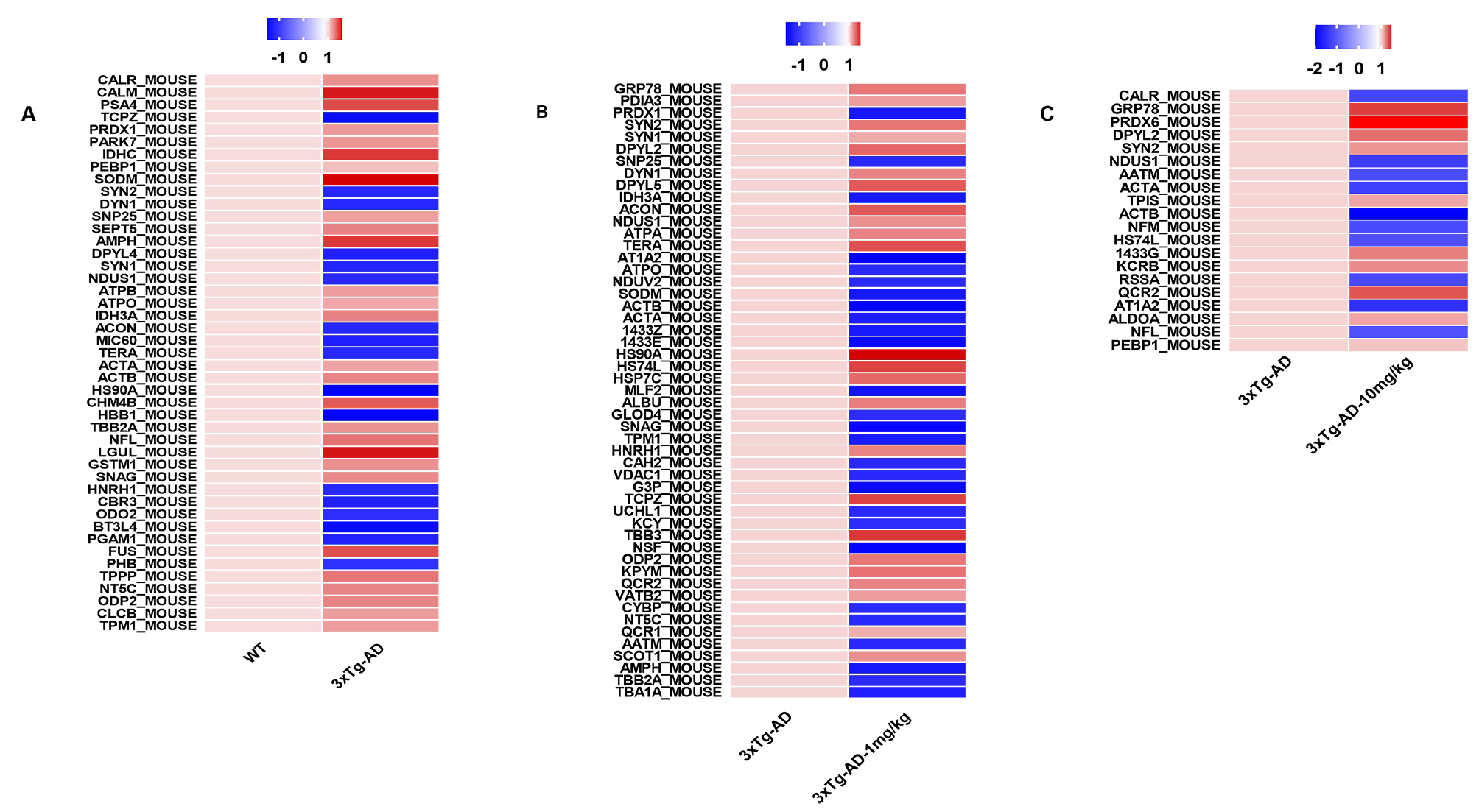

Supplement: Supplementary Figure 2 — Heat maps of the three mouse groups. (A–C) Heat map of the dysregulated proteins in different mouse groups. The rows represent proteins and columns represent groups. Red color indicates high abundance and blue color indicates low abundance. [file Image_2.TIF]

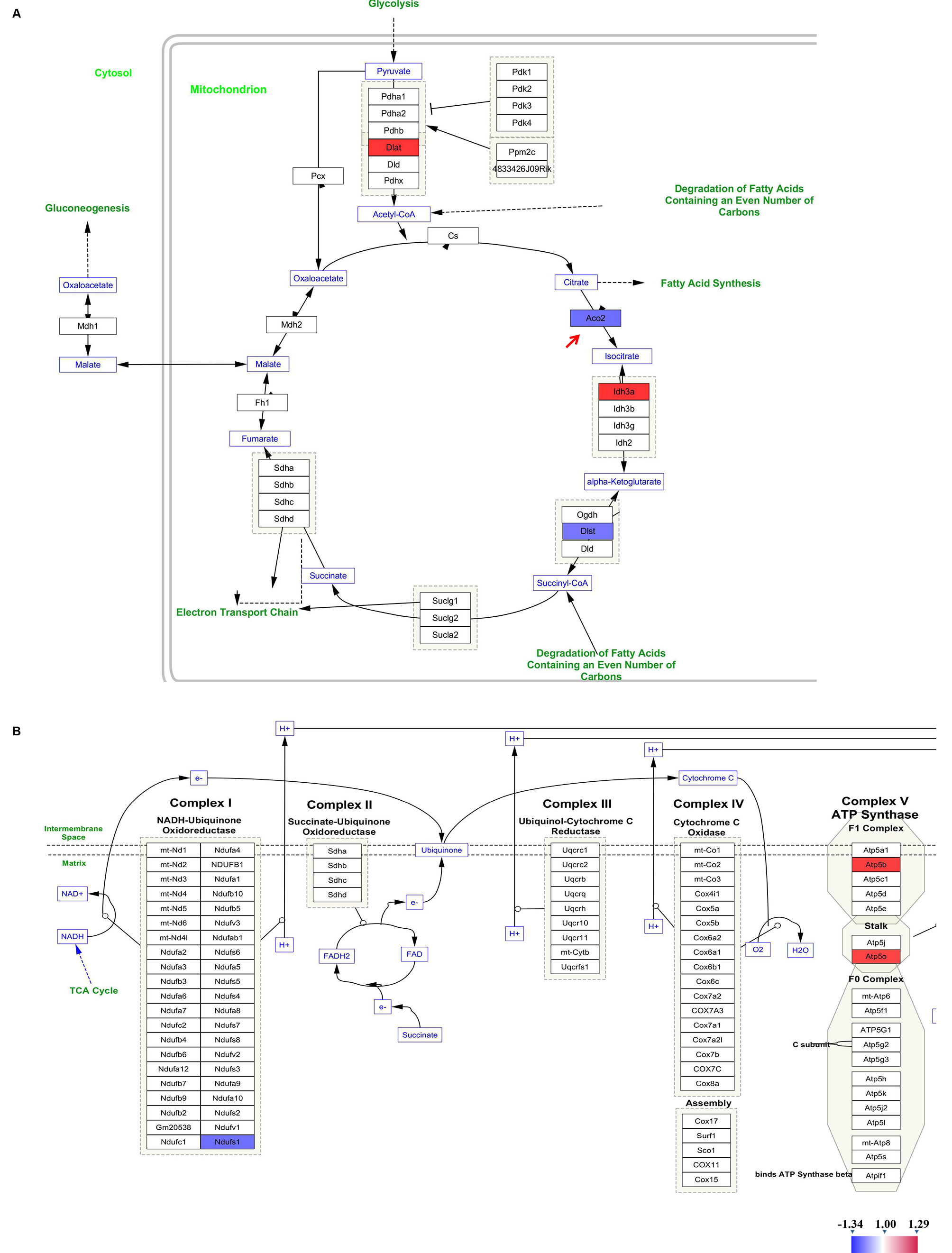

Supplement: Supplementary Figure 3 — The TCA cycle and electron transport chain. (A,B) Comparison of the differentially expressed proteins associated with the TCA cycle and electron transport chain between untreated 3xTg-AD and WT mice and (C,D) between 1 mg/kg/d DAU-treated 3xTg-AD and untreated 3xTg-AD mice. Blue indicates downregulation and red indicates upregulation. [file Image_3.TIF]

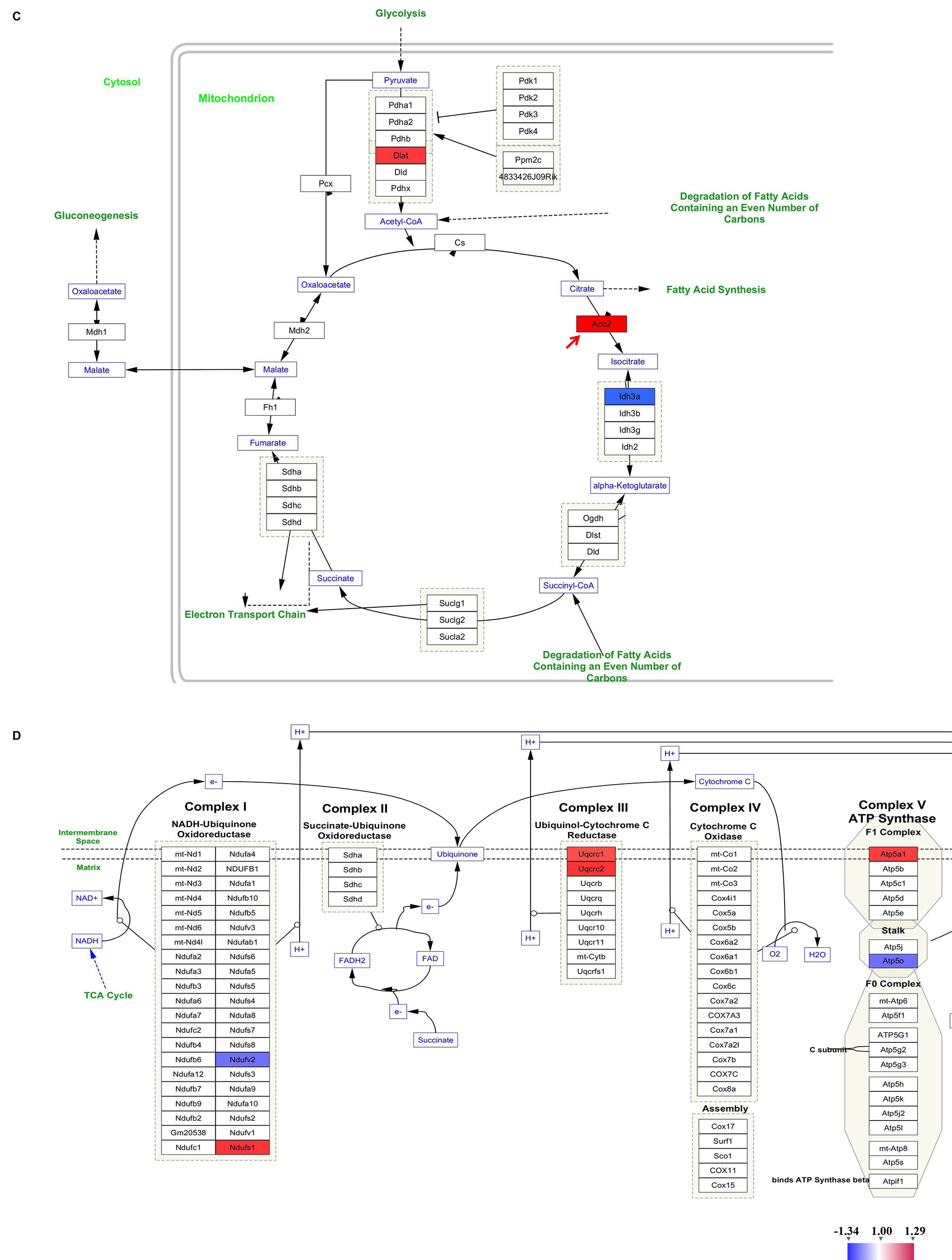

Supplement: Supplementary file 4 [file Image_4.TIF]
